# Knowledge, attitudes, and practices of Florida physicians regarding dengue before and after an educational intervention

**DOI:** 10.1186/s12909-016-0647-8

**Published:** 2016-04-26

**Authors:** Susanne Doblecki-Lewis, Aileen Chang, Renee Jiddou-Yaldoo, Kay M. Tomashek, Danielle Stanek, Leena Anil, Paola Lichtenberger

**Affiliations:** Division of Infectious Diseases, Department of Medicine, University of Miami Miller School of Medicine, 1120 NW 14th Street, #850 (R-21), Miami, FL 33155 USA; Division of General Internal Medicine, George Washington University School of Medicine and Health Sciences, 2150 Pennsylvania Ave NW Room 5-419, Washington, DC 20037 USA; Division of Infectious Diseases, Department of Medicine, Oakland University William Beaumont School of Medicine, Beaumont Health Grosse Pointe, 468 Cadieux Rd, Grosse Pointe, MI 48230 USA; Dengue Branch, Division of Vector-borne Diseases, National Center for Emerging and Zoonotic Infectious Diseases, United States Centers for Disease Control and Prevention, 1600 Clifton Rd, Atlanta, GA 30333 USA; Florida Department of Health, Division of Disease Control and Health Protection, Zoonotic and Vectorborne Disease, 4052 Bald Cypress Way, Bin A12, Tallahassee, FL 32399-1712 USA; Division of Infectious Disease Epidemiology, Bureau for Public Health, West Virginia Department of Health & Human Resources, 350 Capitol St. Rm. 125, Charleston, WV 25301 USA; Division of Infectious Diseases, Department of Medicine, University of Miami Miller School of Medicine, 1120 NW 14th Street, #864 (R-21), Miami, FL 33155 USA

**Keywords:** Dengue, Clinical practice, Physician training, Evaluation, Survey

## Abstract

**Background:**

Failure to recognize and appropriately manage dengue early in the clinical course may result in late initiation of supportive treatment for severe disease. In Florida, travel-related and autochthonous dengue occur and are likely under-recognized. The objective of this study was to evaluate physician knowledge of dengue and its management before and after an educational intervention in Florida.

**Methods:**

From 2012–13 we conducted 14 grand-rounds style lectures on dengue clinical management attended by 413 physicians, and analyzed data from the pre- and post-tests.

**Results:**

Of those attending, 231 and 220 completed the pre-and post-tests, respectively. Overall, the mean pre-test score for knowledge-based questions was 74.3 and average post-test score was 94.2 %, indicating a mean increase of 19.9 % (*P* < 0.0001, 95 % CI 17.7–22.4). Reported confidence in dengue recognition and management also increased. Non-US trained physicians and those who had treated more than ten dengue cases performed significantly better in the pre-test. Post-test scores did not differ by subgroup.

**Conclusions:**

The train-the-trainer approach with grand-rounds style presentations appear to be an effective intervention to improve knowledge of dengue among physicians.

## Background

Dengue is a mosquito-transmitted viral disease that is endemic throughout the subtropics and tropics worldwide. Clinical manifestations range from a mild acute febrile illness to a severe life-threatening disease with a plasma leakage syndrome resulting in hypovolemic shock and hemorrhage [1]. Prior to 1940, dengue outbreaks occurred regularly in Florida including approximately 15,000 cases from 1934–1935. Locally-acquired dengue was not reported again in Florida until 2009–2010 when 88 cases were detected in Monroe County, Florida [2–4]. Since that time, a few sporadic locally-acquired dengue cases have been reported from other Florida counties including an outbreak in Martin County in 2013 [5].

Florida is at risk for dengue outbreaks with locally acquired dengue cases because of its largely non-immune population, an abundance of the mosquito vectors, *Aedes aegypti* and *Aedes albopictus*, and importation of dengue virus via viremic tourists and residents returning from neighboring dengue-endemic areas including Mexico, Central and South American, and the Caribbean. In fact, the majority of dengue cases reported in Florida are travel-associated cases [3]. Florida is a leading reporter of travel-associated dengue cases among the U.S. states and in 2015, Florida was second only to California [6]. Travel-associated cases may result in subsequent autochthonous transmission [2, 5, 7].

While physicians in endemic areas are likely to consider dengue in the differential diagnosis of a patient presenting with an acute febrile illness [8], dengue may not be considered in non-endemic areas, leading to under-diagnosis [9]. In the Key West 2009–2010 outbreak, initial cases were attributed by local physicians to non-specific viral illness and the diagnosis of dengue was not considered [4]. Similarly, in a 1999 dengue outbreak in Laredo, Texas, most patients identified as having a recent dengue infection by serologic diagnostic tests were initially given a clinical diagnosis of non-specific viral syndrome and not dengue [9].

Early recognition of dengue cases and timely initiation of appropriate supportive care is critical because it has been shown to reduce medical complications and mortality among patients with severe dengue [10–14]. As there is no licensed vaccine in the United States to prevent dengue or antiviral medication to prevent the development of severe disease manifestations, educating clinicians on the recognition and clinical management of dengue is an important public health strategy [7]. Educating physicians regarding standard clinical management protocols including how to identify e severe dengue have been demonstrated to reduce the case fatality rate associated with dengue hemorrhagic syndrome [14].

Train-the-trainer interventions, in which a small group of individuals receive in-depth training with the intent to further disseminate information to a broader audience, have been used to train emergency room providers in management of sickle cell disease, improve recognition and treatment of chronic fatigue syndrome, and educate physicians in Puerto Rico in response to a dengue outbreak in 2010 [8, 15, 16]. These interventions allow rapid scale-up of training initiatives and offer promise for rapid dissemination of expert-level content delivered in a local context by physicians within the area.

In response to an increase in travel-associated dengue cases and two outbreaks with several locally acquired dengue cases, the Florida Department of Health (FDOH) in collaboration with University of Miami and the Centers for Disease Control and Prevention (CDC) Dengue Branch developed a train-the-trainer initiative to offer grand rounds-style presentations with continuing medical education (CME) credit for Florida physicians with the goal of increasing early recognition and standardizing treatment of dengue cases. This paper describes the initiative and results from the pre- and post-training evaluation of attendees’ knowledge of dengue.

## Methods

### Study design

This study involved an evaluation of physician knowledge, attitudes, and practices regarding dengue before and after the educational intervention.

### Train-the-trainer symposium

CDC training materials that were used to train physician master trainers in Puerto Rico in 2010 were adapted [8, 17], and used to conduct a one-day, intensive training symposium hosted at the University of Miami. Symposium attendees included a select group of ten physicians from four counties in southern Florida who had prior clinical experience and interest in dengue. The symposium included presentations on the epidemiology, clinical presentation, laboratory diagnosis, reporting requirements, and clinical management of dengue.

### Educational intervention

Following the symposium, a one-hour grand-rounds style presentation was developed using the CDC training materials. The presentation offered an overview of dengue clinical characteristics, diagnosis, monitoring, reporting, and treatment. The physicians attending the training symposium delivered the presentations as part of weekly grand rounds activities at venues within major hospital networks and practices throughout Florida. Participants in the grand rounds sessions were self-selected. One CME credit was offered to attendees of the grand-rounds presentations.

### Evaluation of physician knowledge, attitudes, and practices

To assess physician knowledge and attitudes and practices regarding dengue, a two page, 20-item questionnaire was administered before each presentation and identical questions were repeated after the presentation. The questionnaire assessed participant’s knowledge of dengue virus and its transmission and the epidemiology, diagnosis, reporting, and clinical management of dengue. Questions regarding knowledge and practice were multiple choice questions. The instrument also asked participants for their opinion on the relevance of dengue in their medical practice and confidence in their ability to diagnose and treat dengue patients. These were assessed on a zero to ten scale with zero indicating “no confidence or relevance at all” and ten “very confident/very relevant.” Demographic information including age, years in practice, medical specialty, and location of medical school training was obtained in the pre-test. To preserve confidentiality, pre- and post-tests were not linked. Only those completing the pre-test were asked to complete the post-test.

### Ethics and informed consent

The study protocol was reviewed and approved by the University of Miami Institutional Review Board (study #20120015). A waiver of written informed consent was granted by the Institutional Review Board. A verbal consent script was presented at the beginning of the presentation and attendees were informed that completion of the survey was construed as consent to participate. All attendees were offered the option of not participating.

### Analysis

We conducted a descriptive analysis of participant demographics characteristics, and compared pre- and post-tests findings overall and by demographic groups. The mean percentage of questions correctly answered in the pre- and post-tests were compared using the unpaired student’s *t*-test. For individual items of interest, pre- and post-test responses were compared using the z-test for comparison of proportion of respondents correctly answering the question and the student’s *t*-test with Satterthwaite method for unequal variances for continuous scale responses. Statistical differences in baseline (pre-test) scores were tested using analysis of variance and Tukey’s post-hoc test. The relationship between continuous variables was calculated using Pearson correlation coefficient. Paper questionnaires were completed in-person. Data analysis was conducted using SAS (v. 9.3; SAS Institute Inc., Cary, North Carolina) and graphs were produced in Microsoft Excel (Microsoft Corp., Redmond, WA).

## Results

A total of 413 physicians attended 14 dengue grand-rounds presentations offered in Florida between January 2012 and October 2013. Of these, 231 (55.9 %) completed the pre-test and 220 (53.3 %) also completed the post-test assessment. More than half of all pre-test respondents were reportedly Internal Medicine physicians (42.9 %) or Pediatricians (13.4 %) (Table 1). The majority (68.4 %) of respondents were in practice for 0–5 years while 21.2 % were in practice for more than 10 years. Slightly more than half (54.5 %) of respondents reportedly attended medical school in the United States (U.S.) while 43 % attended medical school in Central America, South America, or the Caribbean. Most (62.3 %) respondents indicated they had never diagnosed a case of dengue; however, nearly one-third of respondents had diagnosed at least one dengue case. Non-U.S. educated physicians were significantly more likely to report having diagnosed at least one dengue case in their career (*p* < 0.0001).

**Table 1:** Demographic characteristics of physicians completing pre-test dengue surveys (*n* = 231)

Medical specialty	*N*	(%)
Internal Medicine	99	42.9
Pediatrics	31	13.4
Dermatology	21	9.1
Infectious Diseases	13	5.6
Emergency Medicine	8	3.5
Internal Medicine/Pediatrics	6	2.6
Family Practice	3	1.3
Other/No Answer	50	21.6
Years in Practice	*N*	(%)
0–5	158	68.4
6–10	16	6.9
> 10	49	21.2
No Answer	8	3.5
Dengue Cases Treated	*N*	(%)
0	144	62.3
1–5	46	19.9
6–10	5	2.2
> 10	18	7.8
No Answer	18	7.8
Medical School	*N*	(%)
In United States	126	54.5
Oustide U.S.	99	42.9
Other/No Answer	6	2.6

Overall, the mean pre-test score for the knowledge-based questions was 74.3 % and the average post-test score was 94.2 %, indicating a mean increase of 19.9 % (*P* < 0.0001, 95 % CI 17.7–22.4) (Table 2). The lowest scoring questions in the pre-test involved identification of clinically significant plasma leakage as the cardinal feature distinguishing severe dengue from dengue (27.3 % correct) and identification of the 1–2 day period after defervescence as the critical phase for development of severe manifestations (40.8 %). High scoring questions included identification of mosquitoes as the vector (98.7 %) and correct management of a patient with warning signs for severe dengue during the critical phase (91.5 %). Scores for all knowledge questions increased from pre-test to post-test assessment.

**Table 2:** Physician survey test results before and after attending training

Test responses mean (SD)	Pre-test	Post-test	*p*-value^a^
	*n* = 231	*n* = 220	
Overall mean number of questions correct	74.3 (14.1)	94.2 (8.7)	<0.001
Knowledge Responses (% who correctly identified)			
Background			
Type of organism that causes dengue is a virus	192/230 (83.5 %)	219/221 (99.1 %)	<0.001
Vector of the disease is a mosquito	227/229 (99.1 %)	221/221 (100 %)	0.16
Infection with one serotype gives lifelong immunity to that serotype	197/224 (83.2 %)	201/215 (93.5 %)	0.05
Intrinsic incubation period within the human	188/226 (83.2 %)	201/218 (92.2 %)	<0.001
Diagnosis			
Cardinal feature distinguishing severe dengue from non-severe cases	60/220 (27.3 %)	198/218 (90.8 %)	<0.001
Timing of the critical phase in dengue	89/218 (40.8 %)	197/215 (91.6 %)	<0.001
Optimal timing to send sample for PCR laboratory diagnosis	132/213 (62.0 %)	178/209 (85.2 %)	<0.001
Management			
Medication used for fever control in a suspected dengue patient	150/223 (67.3 %)	200/215 (93.0 %)	<0.001
Most appropriate treatment for a dengue patient	203/224 (90.6 %)	221/221 (100 %)	<0.001
Management of a suspected dengue patient with warning signs	205/224 (91.5 %)	210/219 (95.9 %)	0.06
Fatality rate with proper recognition and early treatment	133/222 (59.9 %)	194/215 (90.2 %)	<0.001
Timing requirements for reporting to local health department	24/222 (10.8 %)	60/215 (27.9 %)	<0.001
Attitudinal Responses (scale 0–10) Mean (SD)			
Level of confidence recognizing dengue cases (*n* = 216)	4.1 (2.9)	7.0 (2.1)	<0.001
Level of confidence treating dengue cases (*n* = 215)	3.9 (3.2)	7.2 (2.2)	<0.001
Relevance of dengue in their clinical practice (*n* = 215)	6.9 (2.8)	8.3 (1.9)	<0.001

^a^Comparison using paired, Students *t*-test with an alpha set to 0.05

Reported self-confidence in ability to recognize dengue cases increased significantly from pre- to post-test assessment from a mean of score of 4.1 to 7.0, respectively (Table 2). Similarly, confidence in ability to treat dengue cases increased from a mean of 3.9 in the pre-test to 7.2 in the post-test. The assessment of the course relevance to the respondent’s practice increased from 6.9 to 8.3 from pre- to post-test. Greater confidence in recognition and treatment of dengue prior to the presentation were significantly associated with better performance on the pre-test (*r* = 0.34, *p* < 0.001 for recognition; *r* = 0.35, *p* < 0.001 for treatment). Perceived relevance to their own medical practice also demonstrated a weak but significant correlation with pre-test scores (*r* = 0.18, *p* < 0.007).

Average test scores varied by respondent demographic characteristics. Respondents trained at a non-U.S. medical school had higher pre-test scores compared with those educated in the U.S. (76.7 % vs. 72.7 %; *p* = 0.036). Similarly, those who reported diagnosing more than 10 cases of dengue had higher pre-test scores than those who reported having never diagnosed a dengue case (83.8 % vs. 72.0 %; *p* = 0.0003). Respondents who reportedly had never diagnosed a patient with dengue had a significantly greater increase in test score from pre-to post-test compared with those who had diagnosed the most cases (+23.0 % vs. +6.8 %; *p* < 0.0001); post-test scores were not significantly different by number of dengue cases diagnosed. Pre-test and post-test scores did not significantly differ by years in practice.

## Discussion

In our assessment of Florida physicians attending a grand-rounds style presentation on dengue, we found wide variability in baseline knowledge and confidence regarding dengue diagnosis and management. For example, physicians trained at non-U.S. medical schools, in areas where dengue is endemic, reportedly had more experience diagnosing dengue and scored significantly higher on the pre-test than U.S. medical school graduates and those who had never diagnosed a case of dengue. Physician pre-test confidence regarding dengue diagnosis and treatment was also low at baseline, however those with higher pre- test scores tended to report higher levels of confidence.

Knowledge presented in this format appeared to be at a level accessible to practicing physicians with or without prior experience with the disease, and was successful in increasing knowledge and confidence regarding the essential features of diagnosis and treatment of dengue. For example, despite lower baseline knowledge, physicians with no clinical experience with dengue patients and those educated in U.S. medical schools made significantly greater gains from pre-test to post-test than more experienced physicians trained in non U.S. medical schools. In fact, post-test scores did not differ by demographic characteristics and overall test scores were high. In addition, perceived relevance to practice and confidence regarding dengue diagnosis and treatment demonstrated significant gains following the presentation indicating an improved recognition of the importance of dengue among Florida physician attendees.

The finding of differences in dengue knowledge by location of medical school is not unexpected given the small number of dengue cases likely to be encountered by a U.S. medical student during their clinical rotations and the lack of consistent inclusion of tropical medicine education in U.S. medical school curricula [18]. However, physician surveys in areas where dengue is endemic and commonly diagnosed have also demonstrated lack of concordance of practice with available guidelines, lack of recognition of the critical phase of illness, and suboptimal compliance with Department of Health case reporting requirements [8, 19, 20]. Of concern, the recognition of the critical phase of infection in which symptoms may dramatically worsen and correct identification of warning signs were content areas frequently missed in our baseline survey as well as in a recent physician survey conducted in Puerto Rico [8]. Early identification of the warning signs for development of severe dengue can result in more appropriate monitoring and intensive supportive care for more severe symptoms, interventions that have been demonstrated to decrease morbidity and mortality [10–14].

Limitations of this study include non-completion of surveys from some attendees and lack of long-term follow-up to determine retention of knowledge gained. Additionally, while only those participating in the pre-intervention survey were asked to complete the post-intervention survey, the pre- and post-intervention surveys were not individually linked and demographic information was collected only in the pre-intervention survey. Thus, there is the possibility that the individuals completing the post-intervention survey may have differed from those completing the pre-intervention survey. In addition, our study did not investigate if the increase in knowledge and change in attitude demonstrated by respondents led to a change in clinical practice, improved case recognition, or increased testing of suspected cases among attendees. Nevertheless, this assessment and educational program was successful in assessing baseline knowledge of Florida physicians and in disseminating information regarding a locally important disease to many physicians over a short period of time.

## Conclusions

Within the continental United States, Florida is one of the states with both a significant number of imported cases of dengue as well as autochthonous transmission [2]. Early recognition and timely, appropriate treatment of dengue is the cornerstone of public health efforts to mitigate local transmission and optimize patients’ clinical outcomes [7]. Ensuring that Florida physicians know how to recognize potential dengue cases, order appropriate diagnostic tests, and notify public health officials is therefore a priority for any dengue response plan. Further, clinician awareness of the clinical course of dengue including the timing of the critical phase is necessary to offer correct anticipatory guidance for dengue patients and make timely referrals for patients demonstrating warning signs of severe dengue. With increasing international travel and resultant globalization of communicable diseases, there is a need for expanded training for U.S. physicians in the management of tropical diseases [21]. Expanding the traditional medical school curricula to address common emerging tropical diseases affecting travelers and new immigrants may be warranted [22–24]. The train-the-trainer approach and grand-rounds style presentations appear to be an effective intervention to improve knowledge, confidence, and awareness of the importance of tropical diseases that affect Floridians.

## Ethics approval and consent to participate

The study protocol was reviewed and approved by the University of Miami Institutional Review Board (study #20120015). Verbal consent was obtained from each participant prior to survey completion. A waiver of written informed consent was granted by the Institutional Review Board.

## Consent for publication

Not Applicable.

## Availability of data

De-identified data available upon request.

## Abbreviations

CDC: Centers for Disease Control and Prevention
CME: Continuing Medical Education
FDOH: Florida department of health
U.S.: United States
